# Behavioural Methods to Study Cognitive Capacities of Animals

**DOI:** 10.3390/ani13223445

**Published:** 2023-11-08

**Authors:** Lucia Regolin, Maria Loconsole

**Affiliations:** Department of General Psychology, University of Padua, 35131 Padua, Italy; maria.loconsole@unipd.it

Over the past 20 years, the scientific community has witnessed a growing interest in the comparative study of mental capabilities. Animal cognition has become an independent field of interdisciplinary investigation, featuring specialized methodologies and paradigms tailored to diverse animal species. Recently, new approaches have emerged, allowing us access to large numbers of data and offering the possibility of highly reliable analysis with enhanced explicatory power. However, this has often been achieved with the disadvantage of an over-simplification of the behavioural response observed. On the one hand, there are the automated and observer-independent techniques, which allow the collection of vast sets of data but may incur the risk of denaturing the meaning and richness of natural behaviours. On the other hand, there is the study of the neurobiological correlates of mental processes, which requires reference to standardized paradigms, posing constraints on the complexity and ecological validity of behaviour. In contrast to these approaches, more traditional behavioural methods are firmly grounded in species ethology, which is considered crucial in grasping the distinctive qualitative features of behavioural repertoires.

This Special Issue aims to offer a collection of contributions employing behavioural methods to investigate the mental abilities of animals or any other behaving organisms. The Special Issue comprises a series of reviews presenting possible solutions to the key issue of “asking a question” to non-human animals. 

Evidence is reported from groundbreaking research that has contributed novel techniques and approaches to the study of animal minds. Irene Pepperberg offers a review of the model–rival technique, an innovative paradigm that was developed by the author to overcome the limitations of traditional approaches based on conditioning and that, ultimately, allowed the unveiling of complex cognitive abilities in parrots [1]. Thomas Zentall contributes pertinent examples from the current literature, in particular regarding pigeons, that effectively describe the parallelism between human and non-human cognitive abilities, reflecting on the possible existence of shared (i.e., not uniquely human) mechanisms that affect the behaviours of different species [2]. 

Pivotal studies that have made it possible to better describe and comprehend the cognitive and perceptual worlds of animals will be also discussed in detail. Lesley Rogers presents the state of the art of the research on brain lateralization, illustrating the advantages of having a lateralized brain at the levels of both the single individual and the population [3]. Maria Santacà and colleagues tackle the topic of investigating visual illusion as a means of accessing animals’ perceptual worlds, discussing the results achieved so far in this field and critically examining the different paradigms employed with this aim [4]. Andrea Dissegna and colleagues contribute evidence supporting the idea that habituation, previously considered as a sheer single-event learning process, relies, in fact, on some sophisticated learning mechanisms [5]. 

The Special Issue also features research articles that contribute novel knowledge to the field by describing instances of complex (or so-called “higher”) cognitive abilities in non-human subjects. Sigmundson and colleagues test Japanese macaques with a cooperation task in an experimental setting that closely resembles this species’ natural environment and thus allows for the study of macaques’ cognitive abilities while maintaining the social factors that help to shape their behaviours in nature [6]. Alexandra Horowitz and colleagues provide experimental evidence of dogs being able to navigate through a changing environment and discuss how this may represent a way of understanding their sense of themselves [7]. Lisa Horn and colleagues test two corvid species, the azure-winged magpie and the carrion crow, in a prosocial task and use their results as the basis for a discussion on the importance of naturalistic methods and comparisons between different species and experimental paradigms [8]. Greta Baratti and colleagues test spatial and orientation abilities in zebrafish and discuss the relevance of this animal model in comparison with mammalian species [9]. 

Instances of complex cognitive abilities are also discussed, with a particular focus on the importance of accounting for individual variability when evaluating animals’ cognitive performance. Loïc Pougnault and colleagues describe experimental tests for evaluating spontaneous attention in songbirds that could also detect individual variations and attentional characteristics [10]. Marie Pelé and colleagues analyse individual differences in orangutans’ drawing styles, linking them to differences in behavioural styles, states of mind, and motivations [11].

Lastly, new challenges within the study of behaviour, and critical reflections on, and controversies around, the exploitation of current animal models, are presented. Valentina Simonetti and colleagues discuss an example of a non-canonical model for the study of behaviour, showing that plant movement could match some basic features of animal movement in that it is proven to be adaptive, flexible, anticipatory, and goal-directed [12]. Maria Padrell and colleagues discuss the ethical problems posed by invasive research on primates, comparing the present legislation in different countries and reviewing the current evidence of their sophisticated cognitive abilities [13]. Nereida Bueno-Guerra presents a collective metadisciplinary discussion on the current issues within the discipline, including definitions of the key concepts, model species, the use of novel technology and data manipulation, networking, and the impact of sociocultural and ecological factors [14].

Altogether, these contributions offer a multifaceted picture of the current approaches and the challenges that researchers face (and how to overcome some of them) when dealing with the complex topic of animal behaviour and cognition. However, there is still much knowledge on animal minds that is still to be gathered, and the development of new behavioural and ethical methods will allow new insights into the cognitive worlds of animals.
